# Silver Nanoparticle Production by *Ruta graveolens* and Testing Its Safety, Bioactivity, Immune Modulation, Anticancer, and Insecticidal Potentials

**DOI:** 10.1155/2020/5626382

**Published:** 2020-07-24

**Authors:** Hamed A. Ghramh, Essam H. Ibrahim, Mona Kilnay, Zubair Ahmad, Sadeq K. Alhag, Khalid Ali Khan, Ramadan Taha, Fawziah M. Asiri

**Affiliations:** ^1^Research Center for Advanced Materials Science (RCAMS), King Khalid University, P.O. Box 9004, Abha 61413, Saudi Arabia; ^2^Unit of Bee Research and Honey Production, Faculty of Science, King Khalid University, P.O. Box 9004, Abha 61413, Saudi Arabia; ^3^Biology Department, Faculty of Science, King Khalid University, P.O. Box 9004, Abha 61413, Saudi Arabia; ^4^Blood Products Quality Control and Research Department, National Organization for Research and Control of Biologicals, Cairo, Egypt; ^5^Department of Microbiology, National Organization for Drug Control and Research (NODCAR), Cairo, Egypt; ^6^Biology Department, Faculty of Sciences and Arts, King Khalid University, Dhahran Al Janoub, Abha, Saudi Arabia; ^7^Department of Clinical Pathology, Faculty of Veterinary Medicine, Suez Canal University, Ismailia, Egypt; ^8^Department of Biology, Faculty of Science, University of Bisha, Bisha, 511, Saudi Arabia

## Abstract

*Ruta graveolens,* a plant belonging to the family Rutaceae, is traditionally used as a medicinal plant and a flavoring agent in food. This work aimed to prepare silver nanoparticles (AgNPs) using the ethanol extract from *R. graveolens* leaves and test different biological activities as well as insecticidal potentials in the extract and extract prepared AgNPs. Dried and powdered *R. graveolens* leaves were subjected to extraction using ethanol, and this extract was used to synthesize AgNPs. AgNP synthesis was monitored by the change in color, UV spectrophotometry, and electron microscopy (scanning). Fourier transform infrared (FT-IR) spectroscopy was used to monitor the functional groups in the extracts. Immunological, physiological, anticancer, antibacterial, and insecticidal potentials of the extract and its prepared AgNPs were tested. Results showed the ability of the leaf extract to synthesize. SEM examination revealed a spherical shape of AgNPs with a size of 40–45 nm. The extract contained many functional groups as indicated by FT-IR. The extract alone inhibited the growth of normal rat splenic cells, while the extract containing AgNPs stimulated its growth. Extract alone stimulated HeLa cell proliferation and inhibited HepG2 growth, while both cell line growth was inhibited by the extract containing AgNPs. Both the extract and extract with AgNPs were safe on RBCs and did not cause any severe elevation in liver enzymes. The extract alone and with AgNPs showed insecticidal activity against *Culex pipiens*. Our findings suggest that the *R. graveolens* leaf extract, alone or with AgNPs, is biologically safe on animal cells and has antibacterial, insecticidal, and immunomodulation potentials.

## 1. Introduction

Herbal medicines that utilize plant extracts are increasingly used to treat a wide range of diseases, though very little knowledge about the mode of action of these plants is known [1, 2].

*Ruta graveolens* L. (Rutaceae), known in some countries as Sazab and has a common name as rue, is widely distributed in various geographical regions of Afro Asian countries [3]. This plant is cultivated as a decorative plant [4, 5] and is used in traditional medicine for treating many disorders such as hypertension, cramps to hysteria, edema, helminthes, skin conditions, gastrointestinal disorders, and womb diseases [3, 6, 7]. It is also used as a toxin antidote and insect repellent [8]; as a diuretic, antipyretic [5], anticancer [7], antifungal, antibacterial, purgative, antiparasitic, hepatoprotective, antioxidant, and hypotensive agent; and against epilepsy. Ancient Egyptians and early Greeks used rue to improve eyesight and was also used as an anti-inflammatory agent and immune stimulant and for the treatment of skin afflictions and malaria [9, 10]. *Ruta* has been demonstrated to be advantageous for multiple sclerosis treatment [11] as well as hypotensive action [12].

Phytochemical investigations have demonstrated the presence of at least 120 chemical compounds found in all of the different parts of the plant. These include oils, fats, flavonoids, alkaloids, furoquinolines, glycosides, terpenoids, essential oils, steroids, coumarins, sterols, tannins, saponins, phenols, carbohydrates, pyranocoumarins, cardioglycosides, proteins, and amino acids. All parts of the plant contain the active compound, but they are mostly found in leaves [13, 14].

Nobel metal nanoparticles such as silver and gold have obtained increased interest because of their multipurpose applications in several fields such as biology, medicine, and industry [15]. The physiochemical properties of silver nanoparticles (AgNPs) made them get special attention [16]. Nanoparticles can be prepared by chemical and physical methods [17–19], but green synthesis using plants [20, 21], yeast, bacteria, and fungi [22] got more attention because it is nontoxic, clean, and ecofriendly. AgNPs have many biological properties such as anticancer, antimicrobial, antifungal, antiviral, anti-inflammatory [23–26], antiparasite [27], and insecticidal potentials [28]. In addition, silver nanoparticles have been used in the industries such as in paint, detergent [29], clothing [30], and pharmaceutical preparations [31]. Preparation of nanoparticles using a plant extract is valuable due to the ease of preparation methods with low biohazardous contents.

Silver nanoparticles produced by *Ruta graveolens* were shown to have many characteristics that include antibacterial, antifungal [32], dye degradation [33], antiviral [34], and sun protection factor [35].

This study was designed to prepare the *Ruta graveolens* leaf ethanol extract and prepare silver nanoparticles (AgNPs) using this extract. Both the extract and the extract containing AgNPs were tested for their biological safety and as an insecticide.

## 2. Materials and Methods

### 2.1. Preparation of Plant Extracts

*Ruta graveolens* leaves were gathered during the rainy season at Abha, Aseer, Kingdom of Saudi Arabia (KSA), in the month of February 2018. Identification of the plant was kindly done by a taxonomist at the Biology Department, King Abdul-Aziz University, Saudi Arabia. The collected leaves were rinsed with distilled water after extensive washing using tap water. After that, *R. graveolens* leaves were dried in shade at room temperature. Following the method described in [36], the active ingredients in the leaves were extracted. In brief, powder of the dried leaves was prepared by grinding, and 100 g of the obtained powder was mixed with 350 mL of 70% ethanol. The mixture was agitated for 40 h at room temperature and then filtered through a filter paper (Whatman No. 5). Using a rotary evaporator, the obtained flow-throw was dried at 40°C for 3 h. The obtained dried material was about 4 g (semisolid crude extract). From this dried material, 1% acetone and 0.5% dimethyl sulfoxide (DMSO) stock solutions were prepared.

### 2.2. Synthesis of AgNPs Using *R. graveolens* Extract

To an Erlenmeyer flask, 98.5 mL 1 mM AgNO_3_, 0.5 mL Triton X-100 (both are from Sigma-Aldrich), and 1 mL acetone plant extract solution were added to prepare silver nanoparticles (AgNPs) [36]. The flask was incubated at room temperature until the color of the mixture changed (about 24 h).

### 2.3. Characterization of *R. graveolens*-Produced AgNPs

Using a UV-3600 Shimadzu spectrophotometer, the production of AgNPs was monitored at the wavelengths from 200 to 600 nm at 1 nm resolution [36]. The shape and size of the produced AgNPs were characterized using a scanning electron microscope (SEM, JEM-1011, JEOL, Tokyo, Japan) at an accelerating voltage of 90 kV. Fourier transform infrared (FT-IR) spectroscopy (PerkinElmer Spectrum 2000, USA) within the range 600–4000 cm^−1^ at a rate of 16 times, and a clarity of 4 cm^−1^ was used to explore the functional groups in the plant extract alone or with AgNPs.

### 2.4. Antibacterial Activities

#### 2.4.1. Antibacterial Activity Assay

Bacterial strains: Gram-negative (*Escherichia coli*, *Proteus mirabilis*, and *Shigella flexneri*) and Gram-positive (*Staphylococcus aureus*) bacteria were used. These bacteria were kindly supplied by the Microbiology Lab, Biology Department, Faculty of Sciences and Arts, King Khalid University, Dhahran Al Janoub, Saudi Arabia, and were maintained at 4°C on nutrient agar slants. Nutrient agar and nutrient broth (Sigma-Aldrich) were prepared according to the manufacturer's instructions.

#### 2.4.2. Well Diffusion Assay for Antibacterial Activity

The antibacterial potential of the *Ruta graveolens* leaf extract and the extract prepared AgNPs was tested using the agar well diffusion method. Bacterial strains (Gram-negative *Escherichia coli, Proteus mirabilis*, and *Shigella flexneri* and Gram-positive *Staphylococcus aureus*) were inoculated into 15 mL nutrient broth and shaken (225 rpm) overnight at 37°C. Nutrient agar plates were prepared, and 6 mm wells in each plate were punched out. Separate plates were covered with one of the overnight bacterial suspension (50 *μ*L) by spreading using a sterile cotton swap. Plant extract in DMSO and its prepared silver nanoparticles (30 *μ*L each) were aseptically pipetted into the wells of agar plates separately. The positive control (penicillin/streptomycin at 20 units/20 *μ*g, respectively) solution and the negative control (nutrient broth) solution, 30 *μ*L each, were included. Different plates were incubated for 24 h at 37°C, and zones (mm diameter) of inhibition around each well were measured in triplicate [37].

### 2.5. *In Vitro* Effects of *Ruta graveolens* Leaf Extract and Extract Prepared AgNPs on Normal Splenic Cell Proliferation

#### 2.5.1. Splenic Cell Culture Preparation

The test was done according to the procedures described in [38] with little modifications. Splenic single-cell suspension was prepared from healthy adult male Sprague Dawley rats weighing about 280 g. The rat was kindly supplied by the animal house found at King Khalid University. The cells were suspended at a density of 0.05 × 10^6^/mL in a complete RPMI-1640 medium consisting of 10% fetal calf serum (Gibco), penicillin/streptomycin 100 U/100 *μ*g/ml (Gibco), 2 mM L-glutamine (Gibco), 2 mM sodium pyruvate (Seromed), 2% sodium bicarbonate (Seromed), pH 7.2, and HEPES (*N*-2-hydroxyethylpiperazine-*N*-2-ehtanesulfonic acid) buffer (Sigma). The cell culture was performed on a 96-microwell tissue culture plate (Coaster) by adding 100 *μ*L of cell suspension (5000 cells/well) and 100 *μ*L *Ruta graveolens* leaf extract or extract prepared AgNPs at 200, 100, and 50 *μ*g/mL separately.

#### 2.5.2. Study of Cytotoxic/Proliferative Effects

Potentiality of the *R. graveolens* leaf extract and extract prepared AgNPs to kill splenic cells (cytotoxicity) or to induce normal cell division was tested by adding different concentrations of the *R. graveolens* leaf extract and extract prepared AgNPs at final concentrations of 200, 100, and 50 *μ*g/mL to wells containing 5000 cells/well rat splenic cells prepared as mentioned above separately in triplicate. The control culture was prepared by adding 5000 splenic cells/well in 200 *μ*L of the culture medium. All plates were incubated in a CO_2_ incubator (Memmert, GmbH) at 37°C for 72 h. The viability of the cells in all plates was assessed using the Vybrant® MTT cell proliferation assay kit (Thermo Fisher Scientific) according to the manufacturer's instructions.

The percentage increase or decrease in cell number was calculated according to the formula given in [39].

### 2.6. Lytic Effects of *Ruta graveolens* Leaf Extract and Extract Prepared AgNPs on Red Blood Cells (RBCs)

The hemolytic activity which may be found in the *R. graveolens* leaf acetone extract and its biosynthesized AgNPs was tested separately at a final concentration of 200 *μ*g/mL according to the method described in [39] using 10% hematocrit fresh cow red blood cells (RBCs) suspended in the phosphate-buffered saline (PBS, pH 7.4). To 900 *μ*L of prepared RBCs, 100 *μ*L of the acetone *R. graveolens* leaf extract and *R. graveolens* leaf extract prepared AgNPs were added in 1.5 mL tubes separately and incubated for 60 min at 37°C. The positive control was prepared by adding 100 *μ*L of 1.5% Triton X-100 to 900 *μ*L of prepared RBCs (positive control), and the negative control was prepared by adding 100 *μ*L PBS to 900 *μ*L of prepared RBCs. At the end of the incubation, all tubes were centrifuged for 10 min at 2,000 rpm; supernatants were taken from the tubes, and the absorbance of each test was measured at 576 nm. The percentage of hemolysis was determined using the following equation:

(1) $$\begin{array}{c} \%\text{hemolysis}=\left[\frac{\text{absorbance of sample}-\text{absorbance of negative control}}{\text{absorbance of positive control}-\text{absorbance of negative control}}\right]\times 100. \end{array}$$

### 2.7. Acute Cytotoxicity Study of *Ruta graveolens* Leaf Extract and Extract Prepared AgNPs

To test the hepatic toxicity which may be found in the *R. graveolens* leaf extract or extract prepared AgNPs, 5 adult healthy rats (200–250 g) were injected with a single dose regimen of 100 *μ*g/mL of the *R. graveolens* leaf extract or extract prepared AgNPs [39]. The rats were left for 24 h and then sacrificed, and sera were obtained from their blood. Liver function was tested by assaying the level of serum aspartate aminotransferase (AST) colorimetrically according to the Reitman and Frankel method using the Randox kit (UK).

### 2.8. Mosquito Larvicidal Activity

The larval susceptibility test was conducted according the method given by the WHO [40]. Early 4^th^ instar larvae of *Culex pipiens* were treated with various concentrations of *R. graveolens* (1000–5000 ppm) and its prepared AgNPs (50–250 ppm) for 24 h in groups of glass beakers containing 100 mL of tap water. Five replicates of 20 larvae each per concentration, and so in the control, were set up and kept in an environmental chamber at 27°C with a photoperiod of 16 : 8 h light/dark regimen. The larvae were given the usual larval food during these experiments. Larval mortalities were recorded at 24 h after treatment. The dead larvae were identified when they failed to move after being probed by a needle in the siphon or cervical region.

### 2.9. Anticancer Activity Test

The cell lines HepG2 and HeLa were used to test the anticancer potential of the *R. graveolens* leaf extract. The cells were maintained and grown in the supplemented minimal essential medium (MEM), containing fetal calf serum (10%), penicillin/streptomycin (100 U/mL/100 *μ*g/mL), and L-glutamine (2 mM) at 37°C and 5% CO_2_ (CO_2_ incubator). After reaching confluency, the cells were trypsinized (2% trypsin-EDTA) to prepare single-cell suspension. Single-cell suspension was adjusted to 1 × 10^5^/mL, and then 100 *μ*L (10^4^ cells) were plated into each well of the 96-well plate and incubated overnight in a CO_2_ incubator. The medium in the plate was decanted, and 200 *μ*L media containing the *R. graveolens* leaf extract at the concentration of 200 *μ*g/well was added. The plate was incubated for an additional 24 hours in a CO_2_ incubator. The media in wells were replaced with a fresh 100 *μ*L/well culture medium. The viability of the cells was monitored using the Vybrant® MTT cell proliferation assay kit (Thermo Fisher Scientific) according to the manufacturer's instructions [41].

## 3. Statistical Analysis

The study was conducted in a completely randomized design (CRD) in a factorial experiment. All data were statistically analyzed using the analysis of variance (ANOVA), and means were compared by LSD at *P* ≤ 0.05 using the SAS software program, SAS Institute (2006) version 9.3. LC_50_, IC_50_, and IC_95_ were calculated according to the probit analysis program [42]. Computerized log-probit analysis was used to analyze values, regression equations, 95% confidence intervals, and degrees of freedom of the *χ*^2^ goodness of fit tests. Using Abbot's formula [43], the mortality percent was corrected for control mortality.

## 4. Results and Discussion

### 4.1. Characterization of AgNPs

**Figure 1:**
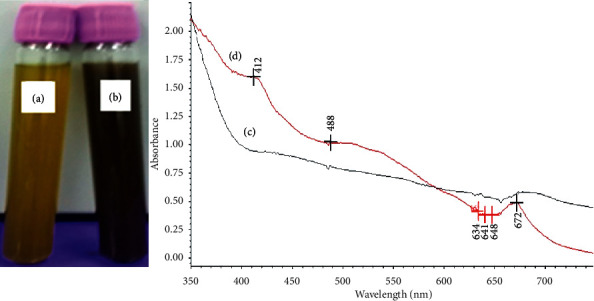
Silver nanoparticle (AgNP) synthesis by the *R. graveolens* leaf extract: (a) extract alone; (b) extract after adding silver nitrate; (c) light absorbance of the extract alone; (d) light absorbance of the extract with AgNPs.

The plant extract was mixed with silver nitrate to synthesize AgNPs. The change in the color of the mixture was an indication of AgNP synthesis where the color of the solution changed from yellow to brown and continued to dark brown (Figures 1(a) and 1(b)). The degree of color change was time dependent that enabled the visual monitoring by observation. This change in color may be assigned to the excitation of the surface plasmon response of AgNPs [44]. Color changes of the solutions are due to some chemical compounds such as alkaloids, flavonoids, saponins, and steroids, and the plant extract may act as a reducing agent that reduced silver ions (Ag^+^) to a silver atom (Ag^0^) by active biomolecules that are found in the plant extract through an enzyme like nitrate reductase. Many enzymes released into the solution have the capability to reduce the silver ions in the form of nanoparticles through agents working as capping molecules such as proteins [44].

**Figure 2:**
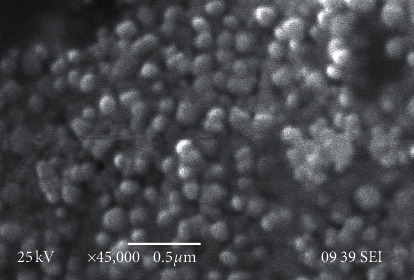
The SEM image showing the spherical silver nanoparticles.

The nanoparticle synthesis was accomplished via one-pot reaction, including the reduction of silver ions using the ethanol extract of *R. graveolens*. Using the UV-Vis absorption, the spectrum of the solution indicated that the surface plasmon resonance that is derived from the silver nanoparticles was around 412–488 nm, which is the specific area of silver nanoparticles (Figures 1(c) and 1(d)). SEM analysis revealed that the prepared AgNPs are almost uniform spherical in shape with a size of 40–45 nm (Figure 2).

**Figure 3:**
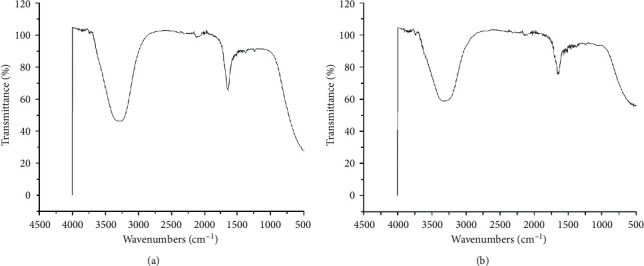
FT-IR spectra of the leaf extract of *R. graveolens* (a) before the addition of AgNO_3_ and (b) after addition of AgNO_3_.

The function of the leaf extract to act as a reducing and capping agent may be due to the domination of several functional groups which were confirmed by FT-IR analysis of the extract containing AgNPs. FT-IR spectroscopy analyses were carried out to recognize the biological entities responsible for capping and stabilizing of AgNPs synthesized using *R. graveolens*. The FT-IR spectra of AgNPs prepared by the leaf extract of *R. graveolens* (Figure 3) showed a vibrational band at 3750 cm^−1^ that was assigned to O-H arising due to alcohols and phenols. The band at 3300 cm^−1^ corresponds to N-H, indicating the presence of primary and secondary amines. The strong stretching vibrational band at 2120 cm^−1^ may be assigned to N=N=N and N=C=N related to azide and carbamide. A weak band at 2000 cm^−1^ may be due to C=C=C and C=C=N, corresponding to allene and ketenimine. The sharp peak at 1630 cm^−1^ may be assigned to C=C, corresponding to alkene. A weak bending band at 1380 cm^−1^ may be assigned to C-H, corresponding to aldehyde and alkane. A weak stretching band at 1250 cm^−1^ indicates C-O and C-N, corresponding to aromatic ester and amine. A stretching vibrational band at 500 cm^−1^ assigned to C-I corresponds to the halo compound [45].

Some works showed that the ethanol extract of the *R. graveolens* plant contained more than 120 compounds such as flavonoids and furoquinolines, coumarins, acridone alkaloids, and essential oils [46]. The components of the *R. graveolens* species are of medical importance because they have a broad range of biological activities which led to some of them being used in medication. Alcoholic extracts of *R. graveolens* have been examined for their antiproliferative effect, using many types of cancer cell lines to test their potentiality as a therapeutic in oncology [47–49].

### 4.2. Antibacterial Activity

The results of testing the antibacterial activity of the *R. graveolens* leaf extract and extract prepared AgNPs against Gram-positive and -negative bacteria are shown in Table 1. Both the plant leaf extract and the extract prepared AgNPs inhibited the bacterial pathogens. Average diameters of inhibition zones produced by plant extract + AgNPs against both Gram-negative and -positive bacteria were bigger than those produced by the plant extract alone.

Previously, many types and sizes of nanoparticles were effectively used to induce the delivery of therapeutic agents [50, 51] to cure bacterial infections in the skin [52]. Also, nanoparticles were used to prevent colonization of bacteria on the surface of medical devices and as an antibacterial agent in food and clothing industries [53]. Because of their unique and nearly known mode of action and antimicrobial properties against Gram-positive and -negative bacteria and the need for developing a new generation of antibiotics, nanoparticles are on the spot as an alternative to traditional antibiotics to bypass the problem of drug resistance.

### 4.3. Cytotoxic/Proliferative Effects

**Figure 4:**
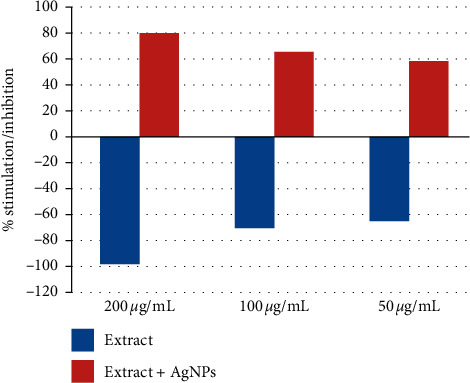
Percent growth stimulation index of normal rat splenic cells after treatment with the leaf extract of *R. graveolens* alone (growth inhibition) and the extract with AgNPs (growth stimulation).

The cytotoxic or stimulatory properties that may be found in *R. graveolens* were studied at different concentrations. The results showed that there were growth inhibitor/cytotoxic effects of the *R. graveolens* leaf extract on normal rat splenic cells, while the extract containing AgNPs showed stimulatory effects on normal splenic cells (Figure 4 and Table 2).

Many constituents of *Ruta graveolens* extracts such as alkaloids, quinolone alkaloids, graveolens, flavonoids, undecyl acetates, and mananones may contribute to its cytotoxic activity. The cytotoxic effects of *Ruta* extracts may be attributed to the induction of DNA damage, causing chromosomal aberrations. They are also reported to be phototoxic, mutagenic, and capable of binding to DNA to induce apoptosis [7]. Flavonoids are reported to be genotoxic and possess oxidant activities. This pro-oxidant activity has been attributed to the apoptotic-inducing property of these flavonoids, and hence implicated in cancer chemoprevention [54]. The study [7] on *Ruta graveolens* found that it produced ROS (reactive oxygen species) and also induced apoptosis, and hence the cytotoxicity activity. Alkaloids were obtained from leaves and stem extracts of rue. Alkaloids are common constituents of other Rutaceae plants [54]. Alkaloids are known to have cytotoxic effects [55]. This explains why the cytotoxic effects decreased with the decrease in the plant concentration added to the normal rat splenic cells. Terpenoids are the largest class of naturally occurring compounds having mainly cytotoxic properties. A large number of terpenoids exhibit cytotoxicity against a variety of cells [56, 57].

The inhibitory effects of the extract decreased with the decrease in the extract concentration. Also, the stimulatory effects decreased with the decrease in the nanoparticle containing extract.

The cytotoxic effects of *R. graveolens* have been demonstrated by other studies [58]. In addition, *R. graveolens* was found to have antitumor activity [7], indicating its toxic behaviors. The stimulatory effects of the extract in the presence of AgNPs may be due to the antagonistic effects of the nanoparticles with the active toxic compounds found in the extract.

### 4.4. Lytic Effects of *Ruta graveolens* Leaf Extract and Extract with AgNP Treatment on RBCs

**Figure 5:**
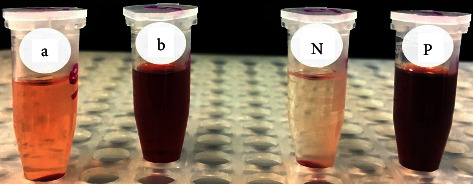
The effect of (a) *R. graveolens* leaf acetone extract alone and (b) its biosynthesized AgNPs on cow RBCs, where N is the negative control and P is the positive control.

The degree of RBC lysis was calculated by comparing the absorbance of the target sample to the positive and negative controls (Table 3). The positive control demonstrated 100% RBC lysis, while the negative control demonstrated 0% RBC lysis. The acetone plant extract containing AgNPs showed 100% RBC lysis while that without AgNPs showed 9.58% RBC lysis (Figure 5 and Table 3). The lysis effect of the extract containing AgNPs may be due to the direct effect of the nanoparticles on the membranes of the RBCs. Other studies referred the hemolytic effects as due to the induced release of oxidative stress products following exposure [59].

### 4.5. Acute Cytotoxicity Study of *R. graveolens* Leaf Acetone Extract and Its Biosynthesized AgNPs

The hepatic toxicity which may be found in prepared extracts was tested in adult healthy rats. The levels of serum aspartate aminotransferase (AST) showed a nonsignificant increase (1.16 fold increase) in the extract alone and the same (1.09 fold increase) in the extract with AgNPs. These results indicate the plant is safe and can be used without any systemic toxic effects. Many authors demonstrated the cytotoxic effects of AgNPs on the liver either *in vivo* [60] or *in vitro* [61]. In our study, the extract containing AgNPs showed no toxic effects on the liver *in vivo*. This may be due to the bioactive compounds found in the extract that abrogated the toxic effects of the nanoparticles on the liver.

### 4.6. Larvicidal Effects of *R. graveolens* Extract and Its Silver Nanoparticles on *Cx. pipiens*

Susceptibility levels of *Cx. pipiens* larvae following treatments with different concentrations of *R. graveolens* and *R. graveolens* synthesized AgNPs against 4^th^ larval instars of *Cx. pipiens* are described in Table 4.

**Figure 6:**
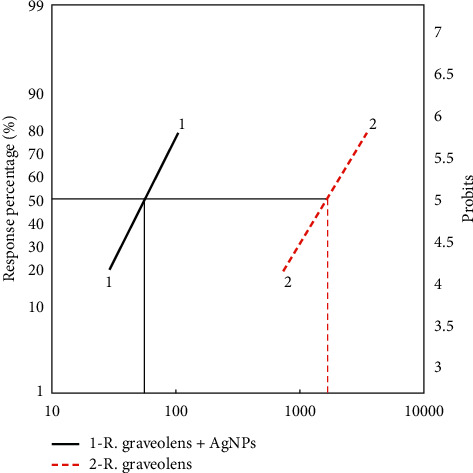
The relationship between concentrations of selected plant extracts and mortality percentage of 4^th^ instar larvae of *Cx. pipiens*. Line 1: *R. graveolens* + AgNo_3_; Line 2: *R. graveolens.*

In general, 31.633–92.857% and 48.98–99.898% larval mortalities were obtained when the 4^th^ instar larvae of *Cx. pipiens* were treated with the effective concentrations of *R. graveolens* alone (1000–5000 ppm) and *R. graveolens* with silver nanoparticles (50–250 ppm), respectively. The results showed a significant positive correlation between the tested concentrations and the percentage of larval death for both the extract alone and the extract containing nanoparticles. Taking the concentration which kills 50% of larvae (LC_50_) and LC_90_ values into consideration, the records showed that the *R. graveolens* with silver nanoparticles caused LC_50_ at 56.002 ppm and LC_90_ at 151.377 ppm, proving that the extract with nanoparticles is more effective than the extract of *R. graveolens* alone that showed LC_50_ at 1673.804 ppm and LC_90_ at 5460.374 ppm, meaning that the extract with nanoparticles has a potency exceeding the extract alone by about 29.888 folds (Figure 6 and Table 5).

The results obtained from this study showed that the effect of the extract of *R. graveolens* and the nanoparticles against the larvae of the *Cx. pipiens* are in accordance with those reported by other works [62, 63].

Kumar et al. [64] reported that 43% mortality was noted for fourth instar larvae of *Culex quinquefasciatus* treated with *S. xanthocarpum*, LC_50_ = 155.29, 198.32, 271.12, 377.44, and 448.41 ppm; LC_90_ = 687.14, 913.10, 1011.89, 1058.85, and 1141.65 ppm, respectively. The bioactivity of the plant as well as AgNPs against the larval instars of *Ae. aegypti* and *An. stephensi* mosquito larvae was determined, and the results of AgNPs showed excellent larvicidal activity of the first, second, third, and fourth instar larvae, which exhibit noticeable effects after 24 or 48 h of exposure at their LC_50_ and LC_90_ values [65].

### 4.7. Effects on Cancer Cells

**Figure 7:**
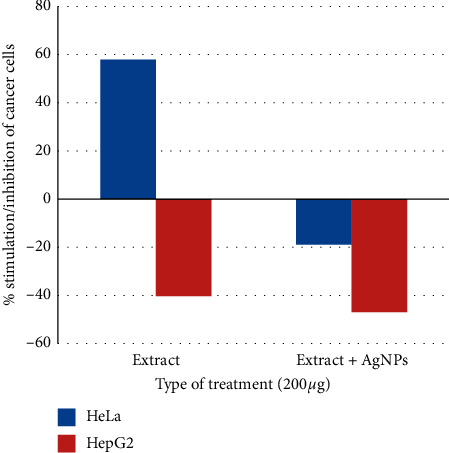
Effects of leaf extracts of *R. graveolens* alone or with AgNPs treatment on HeLa and HepG2 cell line growth stimulation/inhibition.

Effects of the *R. graveolens* extract and extract-generated AgNPs on HeLa and HepG2 cancer cell lines were studied at one concentration. Results showed the ability of the extract alone to diminish the cell growth of the HepG2 cell line but not of the HeLa line. The extract containing AgNPs could lower the growth rate of both cell lines (Figure 7). Similar results were obtained by other studies [66] as they showed that the extract of *R. graveolens* has anticancer effects on some cancer cell lines but not on the HeLa cell line.

Alcoholic extracts of *R. graveolens* have been tested for its antiproliferative power against many types of cancer cells [47–49] and was found to inhibit the growth of these cell lines by many mechanisms. In our study, the *R. graveolens* leaf extract, alone or with AgNPs, inhibited the growth of HepG2.

Generally, the mixing of a plant extract with silver nitrate has led to potentiation. This may be due to the synergy that occurs between the extract and the silver particles during the reduction process, as the compounds present in the extract are connected to the surface of the particles, increasing the strength of their effectiveness [67]; or the strength of the effect may in turn be due to their small size, which make them easy to pass through the wall of the body into the cells where they interfere with the process of dissociation and other physiological processes, and this is consistent with that stated in [68].

## 5. Conclusion

From the above results, we can conclude that the leaf extract of *R. graveolens* could synthesize AgNPs with a size of 40–45 nm. The extract contained functional groups that inhibited the growth of splenic and HepG2 cells and stimulated HeLa cell proliferation. The extract and extract containing AgNPs are safe on RBCs and vital organs. The extract alone and with AgNPs showed insecticidal activity against *Culex pipiens.*

## Figures and Tables

**Table 1:** Antibacterial potentials of the *Ruta graveolens* leaf extract and extract containing AgNPs.

Treatments	Inhibition zone (mm)			
	Bacterial strains			
	*E. coli*	*P. mirabilis*	*S. aureus*	*Sh. flexneri*
Extract	6.33 ± 0.58^c^	6.67 ± 1.15^c^	6.33 ± 0.58^c^	7.33 ± 1.15^c^
Extract + AgNPs	16.33 ± 1.52^b^	15.00 ± 1.00^b^	13.00 ± 1.00^b^	12.67 ± 0.58^b^
Control (positive)	21.67 ± 1.15^a^	20.33 ± 0.58^a^	22.67 ± 1.15^a^	20.00 ± 1.00^a^
Control (negative)	0.00 ± 0.00^d^	0.00 ± 0.00^d^	0.00 ± 0.00^d^	0.00 ± 0.00^d^

Inhibition zones are presented as an average of triplicate ± SD. Means with the same superscript letters indicate nonsignificant differences.

**Table 2:** Effects of the *R. graveolens* leaf extract and extract with AgNP treatment on normal rat splenic cell growth stimulation/inhibition.

(*μ*g/mL)	% of splenic cell growth inhibition/stimulation	
	*R. graveolens* leaf extract	*R. graveolens* leaf extract with AgNPs
200	−98.13 ± 3.50	79.68 ± 3.5
100	−70.22 ± 1.80	65.19 ± 1.80
50	−64.87 ± 1.44	57.50 ± 1.30

% of splenic cell inhibition is expressed as the average of triplicate ± SD.

**Table 3:** Absorbance of lysed RBCs as a result of the *R. graveolens* leaf acetone extract and extract prepared AgNP treatment.

Treatment	Absorbance at wavelength of 576 nm	RBC hemolysis (%)
Plant leaf acetone extract	0.348	9.58
Plant biosynthesized silver nanoparticles (AgNPs)	>3.00	100
Control (negative)	0.067	0
Control (positive)	>3.00	100

**Table 4:** Susceptibility level of *Culex pipiens* larvae to the *R. graveolens* extract and extract containing AgNPs, following continuous exposure for 48 h.

Concentration (ppm)	Observed response %	
	*R. graveolens* extract	*R. graveolens* extract with AgNo_3_
1000	31.633 ± 0.82	48.980 ± 1.18
2000	54.082 ± 0.92	69.388 ± 1.77
3000	72.449 ± 1.11	89.796 ± 2.93
4000	79.592 ± 1.23	96.939 ± 2.88
5000	92.857 ± 2.50	99.898 ± 3.51

**Table 5:** The toxic effect of the *R. graveolens* extract and its AgNPs on the 4^th^ instar larvae of *Cx. pipiens*.

No.	Line name	LC_50_ (ppm)	Lower limit	Upper limit	RR^a^	Slope	LC_90_ (ppm)
1	*R. graveolens* + Ag	56.002	46.515	64.424	29.888	2.968	151.377
2	*R. graveolens*	1673.804	1434.603	1892.906		2.496	5460.374

## Data Availability

The data used to support the findings of this study are included within the article.
